# A pediatric patient with autism spectrum disorder and epilepsy using cannabinoid extracts as complementary therapy: a case report

**DOI:** 10.1186/s13256-020-02478-7

**Published:** 2020-09-22

**Authors:** Juliana Andrea Ponton, Kim Smyth, Elias Soumbasis, Sergio Andres Llanos, Mark Lewis, Wilhelm August Meerholz, Robert Lawrence Tanguay

**Affiliations:** 1 Caleo Health, Suite 200, 1402 8th Ave N.W., Calgary, AB T2N 1B9 Canada; 2 Alberta Children’s Hospital, 2888 Shaganappi Trail N.W., Calgary, AB Canada

**Keywords:** Autism, Cannabidiol extract, Phytocannabinoids, Complementary treatment

## Abstract

**Background:**

The pharmacological treatment for autism spectrum disorders is often poorly tolerated and has traditionally targeted associated conditions, with limited benefit for the core social deficits. We describe the novel use of a cannabidiol-based extract that incidentally improved core social deficits and overall functioning in a patient with autism spectrum disorder, at a lower dose than has been previously reported in autism spectrum disorder.

**Case presentation:**

The parents of a 15-year-old boy, of South African descent, with autism spectrum disorder, selective mutism, anxiety, and controlled epilepsy, consulted a medical cannabis physician to trial cannabis extract to replace seizure medications. Incidentally, at a very low cannabidiol-based extract dose, he experienced unanticipated positive effects on behavioral symptoms and core social deficits.

**Conclusion:**

This case report provides evidence that a lower than previously reported dose of a phytocannabinoid in the form of a cannabidiol-based extract may be capable of aiding in autism spectrum disorder-related behavioral symptoms, core social communication abilities, and comorbid anxiety, sleep difficulties, and weight control. Further research is needed to elucidate the clinical role and underlying biological mechanisms of action of cannabidiol-based extract in patients with autism spectrum disorder.

## Background

Autism spectrum disorder (ASD) is a neurodevelopmental disorder that is characterized by deficits in two major domains: restrictive, repetitive patterns of behavior, interests, or activities; and deficits in social communication and interaction [1, 2]. ASD is associated with a higher incidence of comorbid conditions including attention deficit hyperactivity disorder, anxiety, gastrointestinal disturbances, motor impairments, and epilepsy. Symptoms appear in early childhood and vary in severity leading to a broad range of clinical manifestations [2].

The pathogenesis of ASD is not completely understood [3]. Given its complexity and diverse clinical manifestations, it is believed that the etiopathogenesis of ASD is a combination of genetic, epigenetic, neurobiological, diet, and other environmental factors [4]. Hundreds of genes (*NLGN*, *SHANK3*, *ZNF8034A,* and *UNC13A)* [5, 6] have been linked to ASD, most of which are closely related to the development of the nervous system [1].

There is a myriad of theories that attempt to explain the occurrence of ASD [1, 3, 7], although the two most accepted are impaired synaptic transmission and disruption of neural connectivity. The endocannabinoid system (ECS) has attracted considerable attention as a potential contributor to ASD, as the development of the ECS is essential for regulating synaptic function by inhibiting the release of neurotransmitters from presynaptic neurons [1].

The management of ASD requires individualized, comprehensive treatment. Non-psychopharmacologic interventions (for example, cognitive behavioral therapy) modify disruptive behaviors and improve social communication skills with varying degrees of success. Traditional psychopharmacologic medications target specific ASD core behaviors (for example, repetitive behaviors) and associated behaviors (for example, hyperactivity, aggression, anxiety, and sleep disturbances), but do not treat core social communication deficits [8, 9]. These medications are well known for their substantial side effects. For example, aripiprazole and risperidone, the only two medications approved by the US Food and Drug Administration (FDA) to treat irritability and agitation in ASD, frequently cause somnolence, increased appetite, and weight gain [10]. No other medication has been approved for management of behavioral and/or core ASD symptoms. Challenges with these traditional treatment approaches include barriers to access (economical, geographic), lack of efficacy, and undesirable side effects, which have led many families to seek complementary and alternative medicine (CAM) to augment or replace standard therapy [8]. One of the newest CAM options now being explored in ASD (and, in fact, the wider medical community) is cannabinoids: for example, cannabidiol-based extract (CBE), which is an extract from the cannabis plant, rich in cannabidiol (CBD) [11].

Follow-up of these patients must also be individualized as presentation of the disorder is highly variable. There are no validated questionnaires to accurately assess clinical progress, therefore, conducting an objective clinical assessment of related behavioral and core symptoms is challenging. Despite this, there are tools available for characterizing the overall functionality of patients with ASD, for example, Autism Spectrum Quotient (AQ) adults version [3].

The World Health Organization stated that CBD should not be scheduled with the International Drug Control Conventions because of growing evidence of its medicinal applications [12, 13]. It is imperative for health care providers to understand the minutiae of how cannabinoids interact with the human body and the different forms of cannabinoids that are available for medical use (for example, synthetocannabinoids, phytocannabinoids) [1]. Delta-9-tetrahydrocannabinol (THC) and CBD are the most well-known and studied phytocannabinoids. THC is associated with the impairing psychoactive effects of cannabis, resulting in potentially undesirable side effects (dizziness, anxiety, paranoia, dependency, cognitive impairment, and so on). In contrast, CBD is only minimally psychoactive and not impairing or intoxicating at typically used doses (for example, ≥ 20 mg/kg of CBD referred in the majority of intractable seizures studies) [1, 8, 10, 11].

A multitude of studies have analyzed the use of high-dose CBD extract (~ 20 mg/kg of weight per dose) in the context of intractable seizure treatment [14, 15]. It has been reported that CBD effects are dose-dependent (for example, > 160 mg/day elicits a sedating effect and lower doses have been associated with increased wakefulness) [16]. A few case reports and observational studies have suggested the safety and efficacy of lower dose CBD, for treating behavioral symptoms in ASD [11, 17, 18]. In a prospective study, 188 patients with ASD were treated with lower to medium doses of phytocannabinoids (from 15 mg of CBD three times a day to 300 mg of CBD three times a day), the majority taking 1:20 CBE: 30% CBD to 1.5% THC [19]. This study found that cannabis was well tolerated, safe, and effective in relieving certain ASD symptoms. More research is needed to assess the long-term effects of CBD, as well as optimal dosing, formulation, delivery method, and so on to maximize both safety and efficacy.

This case report describes the clinical presentation of a pediatric, overweight patient with ASD, epilepsy, anxiety, insomnia, and social deficits who benefited clinically with even lower doses of CBE (4 mg of CBD and 0.2 mg of THC twice a day) compared to the ones already studied [19].

## Case presentation

**Fig. 1:**
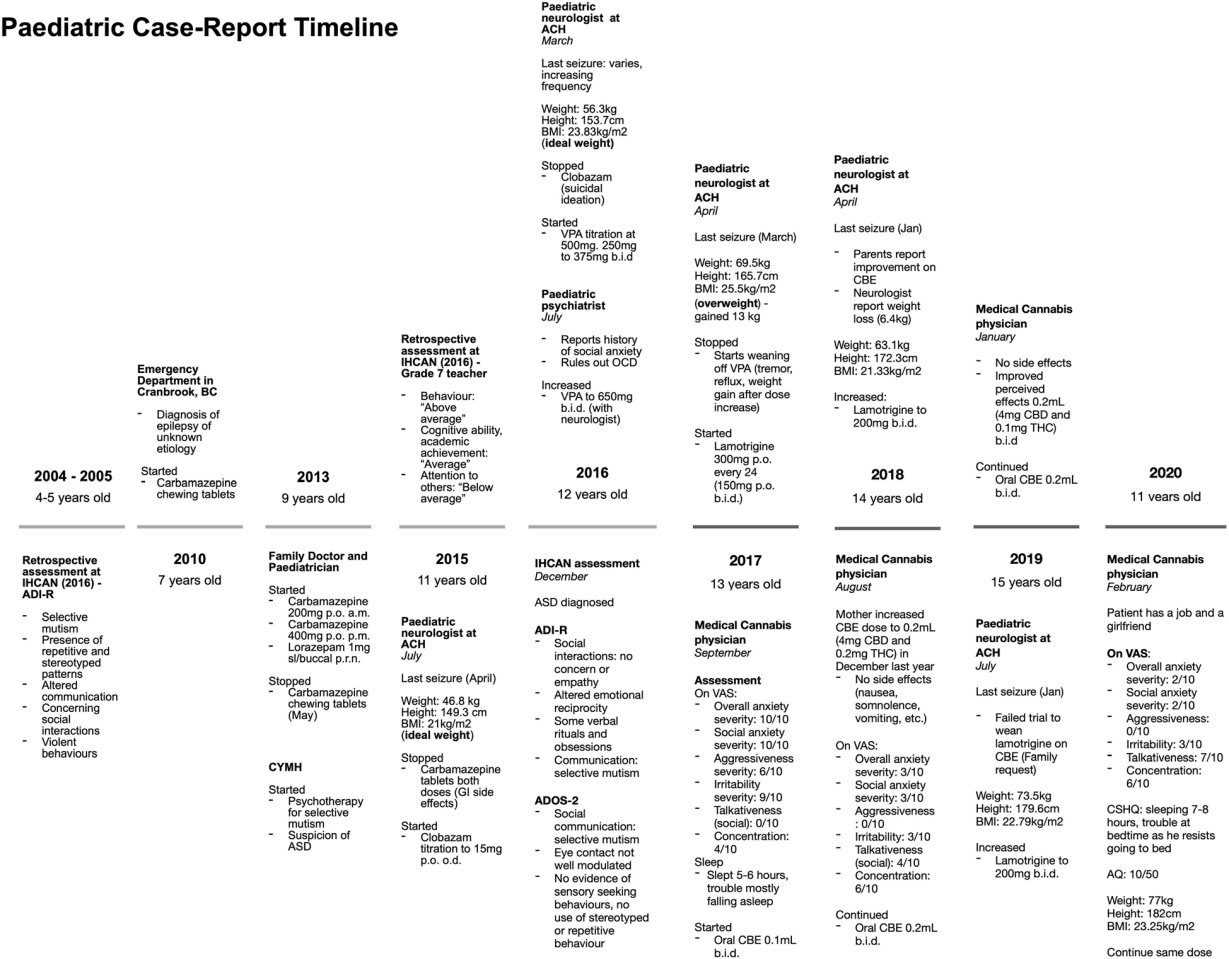
Patient’s timeline depicting important dates and events. *ACH* Alberta Children’s Hospital, *ADI-R* Autism Diagnostic Interview – Revised, *ADOS-2* Autism Diagnostic Observation Schedule 2, *ASD* autism spectrum disorder, *AQ* Autism Spectrum Quotient (Adult), *BC* British Columbia, *BMI* body mass index (calculated by Du Bois method), *CBD* cannabidiol, *CBE* cannabidiol-based extract, CSHQ Children’s Sleep Habits Questionnaire (Abbreviated), *CYMH* Child and Youth Mental Health, *IHCAN* Interior Health Children’s Assessment Network, *OCD* obsessive-compulsive disorder, THC delta-9-tetrahydrocannabinol, *upset stomach* gastrointestinal side effects, *VAS* visual analog scale, *VPA* valproic acid. VAS severity for overall anxiety, social anxiety, aggressiveness and irritability, 0 = least severe, 10 = most severe. VAS for talkativeness, 0 = quiet, 10 = very talkative. VAS for focus, 0 = unfocused, 10 = focused

A 15-year-old boy, of South African descent, is presented with a long-standing history of social and communicative challenges dating back to early childhood, including difficulties in appropriate use of facial expressions, eye contact, and gestures to regulate social interaction (see Fig. 1 for patient’s timeline). He has a history of difficulty in establishing and maintaining relationships, although he has been able to establish some friendships. His mother notes a history of selective mutism dating back to age 3. He has areas of fixated interests and some ritualized behaviors that on assessment were below the threshold for a diagnosis of obsessive-compulsive disorder. In 2016, he was formally diagnosed as having ASD by a specialized organization in British Columbia (BC), the Interior Health Children’s Assessment Network (IHCAN), with supporting evidence from Autism Diagnostic Interview – Revised (ADI-R) and the Autism Diagnostic Observation Schedule 2 (ADOS-2). He does well academically and there are no cognitive concerns. Sometimes he shows aggressive behaviors towards his mother and other relatives.

He was diagnosed as having epilepsy characterized by focal seizures at age 7 at an emergency department service in BC and was subsequently treated by his pediatrician and a pediatric neurologist at the Alberta Children’s Hospital (ACH). He was initially prescribed carbamazepine for seizures which was stopped in 2015 due to side effects (upset stomach), followed by clobazam (stopped in 2016 due to suicidal ideation) and valproic acid (VPA) (stopped in 2017 due to alopecia, tremor, and reflux). The latter also caused a significant weight gain of approximately 13 kg in 1 year, resulting in a calculated body mass index (BMI) with the Du Bois method of 25.5 kg/m^2^. He is currently on lamotrigine for seizures, lorazepam for breakthrough seizures, melatonin for insomnia, riboflavin, ranitidine, magnesium, and orally administered CBE 0.2 mL (4 mg of CBD and 0.1 mg twice a day). No therapy had been tried for behavioral symptoms, although his mother mentioned that VPA and lamotrigine were also prescribed for their effect on mood.

He is currently in psychotherapy at the Child and Youth Mental Health (CYMH) clinic in BC for his selective mutism and anxiety disorder diagnosed by psychiatrists in the same province. He has also had sleep difficulties since 2016. His perinatal history is unremarkable. His birth followed a full-term pregnancy and was uncomplicated except for a required caesarean section due to macrosomia (> 4000 g) and macrocephaly (his mother does not remember the measurement), and subsequent hospitalization for neonatal jaundice. No genetic syndrome was suspected; no genetic testing was ever done. He met expected neurodevelopmental milestones for his age. His mother and grandmother have a history of depression and anxiety. There is other familial history of eating disorders and alcoholism, but no history of genetic syndromes.

In mid-2017, his parents consulted a medical cannabis physician from Caleo Health to assess the suitability of cannabis-based medicines as adjunctive or replacement therapy for seizures. At the time, a physical examination and laboratory findings were normal. A neurologic examination was unremarkable; mental status – awake, alert, cooperative; cranial nerves – normal; motor – normal tone, bulk, strength, and reflexes in upper and lower extremities, proximal and distal, deep tendon reflexes 2+ symmetric. A skin examination was unremarkable, there were no hypopigmented macules, café-au-lait macules, neurofibromas, or axillae ephelides. A long-term (48-hour) electroencephalogram done in 2016 did not record any epileptogenic potentials; magnetic resonance imaging (MRI) showed no intracranial abnormalities and a computed tomography (CT) scan of his head was normal (2015). Laboratory results done mid-2017 were normal: complete blood count and differential, vitamin B12, creatinine, sodium, potassium, calcium, magnesium, total bilirubin, albumin, alkaline phosphatase, aspartate aminotransferase, gamma-glutamyl transferase, alanine aminotransferase, and triacylglycerol lipase. He had low ferritin (11 μg/L) but normal hemoglobin (159 g/L), due to starting a vegetarian diet, which was followed up by the family physician.

He had not had a clinical seizure in 6 months (last seizure in March 2017). Medications at the time of initiation of CBE were: lamotrigine 200 mg twice a day for seizures, lorazepam 1 mg sublingual (SL)/buccal as necessary, infrequently used for seizures, melatonin 6 mg for sleep initiation, riboflavin 400 mg administered orally once daily, magnesium 1 tablet administered orally once daily, and ranitidine 150 mg twice a day. When asked for symptom severity on a visual analog scale (VAS) (0 = least severe, 10 = most severe), his mother reported overall anxiety, social anxiety, aggressiveness, and irritability severity, at 10/10, 10/10, 6/10, and 9/10, respectively. On VAS to assess for talkativeness (0 = quiet, 10 = very talkative) in social situations, the mother reported 0/10. On VAS for concentration (0 = unfocused, 10 = very focused), 4/10 was reported. In regards to sleep, the mother stated he was sleeping approximately 5 to 6 hours, and was having trouble falling asleep. After the initial assessment, his parents gave consent to start therapy and CBE was prescribed (60 mL bottle of 1:20 CBE – 0.001% THC and 0.02% CBD), from CanniMed, with an olive oil carrier. His parents were instructed to administer 0.1 mL twice a day (2 mg CBD and 0.1 mg THC) and increase by 0.1 mL (2 mg CBD and 0.1 mg THC) per dose if no effects were shown to a maximum of 0.5 mL (10 mg CBD and 0.5 mg THC) per dose.

In December 2017, after 3 months of the CBE prescription, his mother increased the dose to 0.2 mL twice a day (4 mg CBD and 0.2 mg THC) as the family noted only mild improvements in anxiety symptoms. In August 2018, a medical cannabis follow-up was conducted. At 0.2 mL twice a day for almost 9 months, our patient’s family reported an improvement of 7 points for overall and social anxiety and irritability, and 6 points on aggressiveness on their respective VAS. Talkativeness improved by 4 points and focus by 2 points. In February 2020, another medical cannabis follow-up was conducted and positive effects were still evident at the same dose. When the mother was asked to complete the Children’s Sleep Habits Questionnaire (CSHQ) Abbreviated, she stated that he slept 7 hours and only had trouble falling asleep in his own bed as he resists going to bed. No side effects were reported (nausea/vomiting, diarrhea, headaches, euphoria, feeling high, anxiety, panic attacks, palpitations, somnolence during the day or drowsiness). Laboratory results remained normal and low ferritin was corrected. He began to initiate and reciprocate conversations with acquaintances he had previously been unable to speak to (for example, doctors, community members). He became more motivated and energetic, starting his own vegetarian diet and exercise programs, ultimately losing 6.4 kg after starting CBE for a calculated BMI of 21.33 kg/m^2^. He was able to start his first part-time job helping customers and interacting with them. He was instructed to fill out the self-administered Adult AQ which resulted in a normal score of 10 as shown in Table 1. His mother stated he now also has a girlfriend. Recently, his mother started weaning him off CBE to go on a trip and noted an immediate change. He became more irritable and aggressive.

**Table 1:** Adult Autism Spectrum Quotient score

Item	Patient’s score	Maximum score
Social skills	2	10
Attention switching	2	10
Attention to detail	3	10
Communication	1	10
Imagination	2	10
Total	10	50

In a study by Baron-Cohen *et al*. [20], control individuals showed a mean score of 16.5 while individuals with Asperger syndrome and high-functioning autism showed a mean score of 35

In discussion with their neurologist, the family decided to wean lamotrigine while remaining on CBE (0.2 mL twice a day – 4 mg CBD and 0.2 mg THC). Unfortunately, there was a recurrence of seizures and lamotrigine was titrated back to the full 200 mg twice a day dose.

Currently, our patient remains on the same medication as mentioned above, as well as low dose of CBE. He has maintained the positive effect on his behavioral symptoms, anxiety, sleep, and social deficits on CBE 1:20 ratio, 0.2 mL twice a day (4 mg CBD and 0.2 mg THC) and no side effects have been reported.

## Discussion

This case demonstrates the benefit of a lower than previously studied CBE dose for core social communicative and behavioral ASD symptoms, as well as improvements in co-occurring anxiety, sleep dysregulation, and weight, which led to substantial improvements in both our patient’s and his family’s quality of life and daily functioning. There was partial response at the initial dose of 0.1 mL twice a day (2 mg CBD and 0.1 mg THC) and a dramatic response at 0.2 mL twice a day (4 mg CBD and 0.2 mg THC). Seizures recurred when conventional anti-epileptic medication (lamotrigine) was weaned while on the CBE at the 0.1 mL twice a day dose (low doses), reiterating CBE at this dose did not have any significant anti-epileptic effect; lamotrigine has not been weaned while on the 0.2 mL twice a day (4 mg CBD and 0.2 mg THC) dose of CBE. Typically, significant higher CBD doses are needed for seizure control (> 20 mg/kg per day) [14, 15].

The symptom improvement occurred within a 6-month period following the initiation of CBE treatment, during which time there were no new additions or significant alterations of/to any concurrent medications or therapies that could otherwise explain the improvements in symptomatology. It remains unclear whether the CBE directly modified the core ASD symptoms in some way, or whether the impact of CBE was secondary to its positive effects on comorbid conditions, namely anxiety and/or sleep dysregulation, which were producing or exacerbating underlying ASD behaviors. We must also consider there are limitations inherent in the method used to assess his clinical improvement, as the VAS and the AQ are not yet validated. These measures were chosen by default, as no scale is currently validated to assess clinical progress [3]. Seizures recurred at the initial 0.1 mL twice a day (2 mg CBD and 0.1 mg THC) dose of CBE. In addition to the fact that seizures were well controlled prior to starting CBE, the recurrence of seizures on the initial 0.1 mL twice a day dose of CBE, a dose at which symptoms were already starting to improve, suggests that improvements in ASD symptoms were not related to improvements in epilepsy control; the anti-seizure properties of CBD alone are unlikely to be the predominant mechanism responsible for the improvements in this patient’s ASD symptoms.

The ECS is a unique biological system that is present in the majority of body tissues. It plays an important role in cellular processes at the early stages of development [21]. The ECS is an essential regulatory system of the central nervous system that modulates both neurotransmission and synaptic plasticity. It is also involved in emotional and social functioning, and cognition [1, 21]. There is evidence that the ECS is underdeveloped in ASD [1, 22]. CBD may be treating core symptoms in ASD by interacting with the ECS to boost function in one way. CBD may increase the availability of the endogenous cannabinoids, anandamide (AEA), by directly inhibiting one of its degrading enzymes, that is, fatty amide acid hydrolase (FAAH) [1, 23, 24]. Wei *et al.* demonstrated that selective inhibition of FAAH in BTBR animals, increased AEA activity [25]. Further to this, a case–control study by Karhson *et al*. assessed AEA concentrations in ASD (*n* = 59) versus controls, and found lower AEA concentrations associated with ASD [26].

High-dose CBD has been studied for seizures and has been approved by the FDA (Epidiolex) for the treatment of intractable epilepsy [14, 15, 27, 28], but there remains a lack of evidence for the use of phytocannabinoids in ASD [11]. Only a few low-powered studies address the clinical efficacy of cannabinoids for such symptoms, and there are no established recommendations for its use in ASD treatment [5, 6]. The majority of published studies for ASD either involve synthetic cannabinoids [11, 17, 18] or synthetic enzyme-inhibitors [25, 29]. Only a few studies offer evidence for the use of phytocannabinoids in ASD. An observational study by Bar-Lev Schleider *et al.* provided valuable information on safety and efficacy, but the study design was insufficient to draw strong conclusions on standard clinical care [19]. Clinicaltrials.gov lists an ongoing randomized trial comparing different phytocannabinoid extracts in the setting of behavioral symptoms, but results are not yet available [30]. Therefore, this case report is rare as it documents observed effects of CBE in ASD-related symptoms as opposed to other forms of cannabinoids (for example, nabilone, dronabinol, and nabiximols).

From a clinical perspective, the use of CBD-based products to treat neuropsychiatric symptoms must be done only after appropriate education and informed discussion with families, including consideration of risks and benefits of CBD compared to other available treatment options, and with vigilant monitoring.

Research into the role of cannabinoids in treating ASD symptoms and associated behaviors is in its infancy. Although there is an increasing amount of evidence providing biological plausibility for the use of CBD in treating ASD [1, 5, 25, 26, 31], further research is essential to better understand the effects of phytocannabinoids on neurobiological pathways and their impact on behavior and brain function. Rigorous, controlled clinical trials are needed to further establish safety, especially long-term safety, optimal dosing, and efficacy, including further delineation of the effect of CBE on core versus associated ASD symptoms. Until sufficient, supportive evidence is found, CBE remains an unproven alternative treatment and should not replace conventional evidence-based treatments for children with autism. However, the unexpected and significant benefits of CBE in this case report highlight the urgent need and potential benefits of continuing to pursue research in this area.

## Conclusion

While there is a lack of strong evidence to support the use of CBE in ASD, this case report provides the first insight about lower than previously reported doses of phytocannabinoids in the form of CBE, which may benefit ASD-related behavioral and core social symptoms, as well as anxiety, sleep disturbances, and weight. We encourage scientists and clinicians to pioneer placebo-controlled studies to validate the clinical efficacy of very low doses of CBE in a larger cohort.

## Data Availability

The dataset generated and analyzed during this case report are available in Netcare (Alberta’s public Electronic Health Record used to store patient information).

## Abbreviations

AEA: Anandamide
ASD: Autism spectrum disorder
ECS: Endocannabinoid system
FDA: Food and Drug Administration
CAM: Complementary and alternative medicine
CBD: Cannabidiol
CT: Computed tomography
CYMH: Child and Youth Mental Health
FAAH: Fatty amide acid hydrolase
THC: Delta-9-tetrahydrocannabinol
CBE: Cannabidiol-based extract
IHCAN: Interior Health Children’s Assessment Network
MRI: Magnetic resonance imaging
SL: Sublingual
ADI-R: Autism Diagnostic Interview – Revised
ADOS-2: Autism Diagnostic Observation Schedule 2
ACH: Alberta Children’s Hospital
VPA: Valproic acid
BMI: Body mass index
BC: British Columbia
VAS: Visual analog scale
CSHQ: Children’s Sleep Habits Questionnaire
AQ: Autism Spectrum Quotient

## References

[1] Zou M, Li D, Li L, Wu L, Sun C. Role of the endocannabinoid system in neurological disorders. Int J Dev Neurosci. 2019;76:95–102. 10.1016/j.ijdevneu.2019.03.002 30858029

[2] American Psychiatric Association. DSM-5 Diagnostic Classification. Diagnostic and Statistical Manual of Mental Disorders. American Psychiatric Association; 2013 [cited 2019 Nov 6]. Available from: https://psychiatryonline.org/doi/10.1176/appi.books.9780890425596.x00DiagnosticClassification.

[3] Lai MC, Lombardo MV, Baron-Cohen S. Autism. Lancet. 2014;383(9920):896–910. 10.1016/S0140-6736(13)61539-1 24074734

[4] Geschwind DH. Genetics of autism spectrum disorders. Trends Cogn Sci. 2011;15(9):409–16. 10.1016/j.tics.2011.07.003 PMC3691066 21855394

[5] Jamain S, Quach H, Betancur C, Råstam M, Colineaux C, Gillberg IC, *et al.* Mutations of the X-linked genes encoding neuroligins NLGN3 and NLGN4 are associated with autism. Nat Genet. 2003;34(1):27–9. 10.1038/ng1136 PMC1925054 12669065

[6] Nguyen QA, Horn ME, Nicoll RA. Distinct roles for extracellular and intracellular domains in neuroligin function at inhibitory synapses. Elife. 2016;5:e19236. 10.7554/eLife.19236 PMC5098909 27805570

[7] Yenkoyan K, Grigoryan A, Fereshetyan K, Yepremyan D. Advances in understanding the pathophysiology of autism spectrum disorders. Behav Brain Res. 2017;331:92–101. 10.1016/j.bbr.2017.04.038 28499914

[8] Weissman L. Autism spectrum disorder in children and adolescents: Overview of management. In: Augustyn M, Patterson MC, Torchia MM, editors. UpToDate [Internet]. Waltham (MA): UpToDate Inc.; 2019 [updated 2019 Dec 19; cited 2019 Sep 19]. Available from: https://www.uptodate.com/contents/autism-spectrum-disorder-in-children-and-adolescents-overview-of-management?search=autismspectrumdisorder&source=search_result&selectedTitle=1~146&usage_type=default&display_rank=1.

[9] Posey DJ, McDougle CJ. Pharmacotherapeutic management of autism. Expert Opin Pharmacother. 2001;2(4):587–600. 10.1517/14656566.2.4.587 11336609

[10] Goel R, Hong JS, Findling RL, Ji NY. An update on pharmacotherapy of autism spectrum disorder in children and adolescents. Int Rev Psychiatry. 2018;30(1):78–95. 10.1080/09540261.2018.1458706 29693461

[11] Weissman L, Hodges HK. Autism spectrum disorder in children and adolescents: Complementary and alternative therapies. In: Augustyn M, Patterson MC, Torchia MM, editors. Waltham (MA): UpToDate Inc.; 2019 [updated 2020 Feb 05; cited 2019 Sep 19]. Available from: https://www.uptodate.com/contents/autism-spectrum-disorder-in-children-andadolescents-complementary-and-alternative-therapies?search=Autism spectrum disorder in children and adolescents: Complementary and alternative therapies&source=.

[12] Whiting PF, Wolff RF, Deshpande S, Di Nisio M, Duffy S, Hernandez AV, *et al.* Cannabinoids for Medical Use: A Systematic Review and Meta-analysis. JAMA. 2015;313(24):2456–73. 10.1001/jama.2015.6358 26103030

[13] World Health Organization. WHO Expert Committee on Drug Dependence: Fortieth Report [Internet]. 2018 [cited 2020 Jan 29]. Available from: https://apps.who.int/iris/bitstream/handle/10665/279948/9789241210225-eng.pdf?ua=1#:~:text=The fortieth meeting of the WHO, cannabis and its component substances.

[14] Devinsky O, Cross JH, Laux L, Marsh E, Miller I, Nabbout R, *et al.* Trial of Cannabidiol for Drug-Resistant Seizures in the Dravet Syndrome. N Engl J Med. 2017;376(21):2011–20. 10.1056/NEJMoa1611618 28538134

[15] Devinsky O, Patel AD, Cross JH, Villanueva V, Wirrell EC, Privitera M, *et al.* Effect of Cannabidiol on Drop Seizures in the Lennox–Gastaut Syndrome. N Engl J Med. 2018;378(20):1888–97. 10.1056/NEJMoa1714631 29768152

[16] Babson KA, Sottile J, Morabito D. Cannabis, Cannabinoids, and Sleep: a Review of the Literature. Curr Psychiatry Rep. 2017;19(4):23. 10.1007/s11920-017-0775-9 28349316

[17] Kurz R, Blaas K. Use of dronabinol ( delta-9-THC ) in autism: A prospective single-case-study with an early infantile autistic child. Cannabinoids. 2010;5(4):4–6.

[18] Kruger T, Christophersen E. An Open Label Study of the Use of Dronabinol (Marinol) in the Management of Treatment-Resistant Self-Injurious Behavior in 10 Retarded Adolescent Patients. J Dev Behav Pediatr. 2006;27(5):441.

[19] Bar-Lev Schleider L, Mechoulam R, Saban N, Meiri G, Novack V. Real life Experience of Medical Cannabis Treatment in Autism: Analysis of Safety and Efficacy. Sci Rep. 2019;9(1):200. 10.1038/s41598-018-37570-y PMC6336869 30655581

[20] Baron-Cohen S, Wheelwright S, Skinner R, Martin J, Clubley E. The autism-spectrum quotient (AQ): evidence from Asperger syndrome/high-functioning autism, males and females, scientists and mathematicians. J Autism Dev Disord. 2001;31(1):5–17. 10.1023/a:1005653411471 11439754

[21] Richardson KA, Hester AK, McLemore GL. Prenatal cannabis exposure - The “first hit” to the endocannabinoid system. Neurotoxicol Teratol. 2016;58:5–14. 10.1016/j.ntt.2016.08.003 27567698

[22] Lafourcade M, Larrieu T, Mato S, Duffaud A, Sepers M, Matias I, *et al.* Nutritional omega-3 deficiency abolishes endocannabinoid-mediated neuronal functions. Nat Neurosci. 2011;14(3):345–50. 10.1038/nn.2736 21278728

[23] Schwarz R, Ramer R, Hinz B. Targeting the endocannabinoid system as a potential anticancer approach. Drug Metab Rev. 2018;50(1):26–53. 10.1080/03602532.2018.1428344 29390896

[24] Philpott HT, O’Brien M, McDougall JJ. Attenuation of early phase inflammation by cannabidiol prevents pain and nerve damage in rat osteoarthritis. Pain. 2017;158(12):2442–51. 10.1097/j.pain.0000000000001052 PMC5690292 28885454

[25] Wei D, Dinh D, Lee D, Li D, Anguren A, Moreno-Sanz G, *et al.* Enhancement of Anandamide-Mediated Endocannabinoid Signaling Corrects Autism-Related Social Impairment. Cannabis Cannabinoid Res. 2016;1(1):81–9. 10.1089/can.2015.0008 PMC5549436 28861483

[26] Karhson DS, Krasinska KM, Dallaire JA, Libove RA, Phillips JM, Chien AS, *et al.* Plasma anandamide concentrations are lower in children with autism spectrum disorder. Mol Autism. 2018;9(1):1–6. 10.1186/s13229-018-0203-y PMC5848550 29564080

[27] Food and Drug Administration. Medication Guide - Epidiolex. In Carlsbad (CA): Greenwich Biosciences Inc.; 2018 [cited 2019 Nov 23]. p. 30. Available from: https://www.accessdata.fda.gov/drugsatfda_docs/label/2018/210365lbl.pdf.

[28] Thiele EA, Marsh ED, French JA, Mazurkiewicz MB, Benbadis SR, Joshi C, *et al.* Cannabidiol in patients with seizures associated with Lennox-Gastaut syndrome (GWPCARE4): a randomised, double-blind, placebo-controlled phase 3 trial. Lancet. 2018;391(10125):1085–96. 10.1016/S0140-6736(18)30136-3 29395273

[29] Gould GG, Seillier A, Weiss G, Giuffrida A, Burke TF, Hensler JG, *et al.* Acetaminophen differentially enhances social behavior and cortical cannabinoid levels in inbred mice. Prog Neuro-Psychopharmacology Biol Psychiatry. 2012;38(2):260–9. 10.1016/j.pnpbp.2012.04.011 PMC3389197 22542870

[30] ClinicalTrials.gov [internet]. United States: Bethesda (MA): US National Library of Medicine; 2000 - . Identifier NCT02956226, Cannabinoids for Behavioral Problems in Autism Spectrum Disorder: A Double Blind, Randomized, Placebo-Controlled Trial with Crossover; 2016 Nov 6 [cited 2019 Sep 19]. Available from: https://www.clinicaltrials.gov/ct2/show/study/NCT02956226?show_desc=Y#desc.

[31] Mazahery H, Stonehouse W, Delshad M, Kruger MC, Conlon CA, Beck KL, *et al.* Relationship between long chain n-3 polyunsaturated fatty acids and autism spectrum disorder: Systematic review and meta-analysis of case-control and randomised controlled trials. Nutrients. 2017;9(2):1–32. 10.3390/nu9020155 PMC5331586 28218722

